# Supplementary material for: The sensitivity of transcriptomics BMD modeling to the methods used for microarray data normalization
Source: PLoS One. 2020 May 15;15(5):e0232955. doi: 10.1371/journal.pone.0232955 (PMC7228135; doi:10.1371/journal.pone.0232955)
Supplement: S1 File — (PDF) [file pone.0232955.s002.pdf]

Supplemental online file

The sensitivity of transcriptomics BMD modeling to the methods used for microarray data normalization

Mezencev R. and Auerbach S.

1. Supplemental methods

1.1 Transcriptomic BMD modeling parameters:

- Prefilter: Williams' trend test; p-value cutoff: 0.05; Filter out control genes (probes starting AFFX...); Use Fold Change Filter 1.50
- Benchmark Dose Analyses: Continuous models Hill (v. 2.18), Power (v. 2.19), Linear, Poly 2 (v. 2.21), Exp2, Exp3, Exp4, Exp5 (v. 1.11); Restrict Power ≥1; Confidence Level=0.95; Constant Variance; Maximum iterations=250; BMR=1 SD
- Best Model Selection: Lowest AIC; Flag Hill model with k <1/3 of Lowest Positive Dose and if this model is best, select next best model with p>0.05

1.2 Functional classifications parameters:

Gene Ontology Analyses: GO Categories:Biological process (or Select Pathway: REACTOME)
Remove BMD>highest dose from category descriptive statistics
Remove BMD with p-value < 0.1
Remove Genes with BMDU/BMDL>40
Eliminate Gene Set Redundancy
Identify Conflicting Probesets: correlation cutoff: 0.5
Most sensitive pathway was identified as pathway with lowest median BMD value when contained at least 3 genes that passed probe-level BMD filtering and when the proportion of these genes in the pathway reached at least 5%.

2. Supplemental tables

Supplemental Table 1 Comparison of methods used for hybridization of the raw Affymetrix Rat Genome 230 2.0 Array data in this study

	Background estimation	Normalization	Summarization	Other
GCRMA	Model of PM and MM signals that include sequence-dependent non-specific binding and optical noise for each probe pair.	Quantile normalization	Robust multiarray linear model fit using Median Polish	-Employs MM intensities to correct background<br><br>-Results on log2 scale
RMA	Convolution model of the observed PM probe signal as a sum of signal and background. Multiplicative error. Single global background used to adjust all intensities.	Quantile normalization	Robust multiarray linear model fit using Median Polish	-Does not use MM intensities<br><br>-Results on log2 scale
MAS 5.0	Weighted average of lowest 2% of signals from 16 equal rectangular regions. Weighting reflects distance of 16 averages from a particular probe.<br>PM signals adjusted by MM intensities to correct for non-specific binding.<br>Multiplicative error.	Linear scaling using the trimmed mean that excludes the highest and lowest 2% of the data	Robust single-array method using one step Tukey biweight of background corrected intensities	-Employs MM intensities<br><br>-Results on linear scale
MAS 5.0_noA	As in MAS 5.0	As in MAS 5.0	As in MAS 5.0	-Probes with only “Absent” calls across all specimens are removed<br><br>-Results on linear scale
PLIER*	PM-MM method. Mixed error model.	Quantile normalization	Multiplicative model fitted to the PM-MM values, with array and probe as effects. An M-estimator is used that assess goodness of fit by the residuals generated from a generalized log transformation based on the PM and MM	-Employs PM and MM intensities.<br><br>-Results on linear scale
PLIER16*	As in PLIER	As in PLIER	As in PLIER	-As in PLIER<br>-16 is added to normalized signal intensities
PLIER16_noA*	As in PLIER16	As in PLIER16	As in PLIER16	-As in PLIER16<br><br>-Probes with only “Absent” calls across all specimens are removed

* Relates to PLIER as specifically implemented in this study

Supplemental table 2 Median lowest BMD (BEPOD) and BMDL (BEPOD/L) values [mg/kg-day] determined by toxicogenomic BMD modeling for different normalization methods and different gene sets

Collection of gene sets	**GCRMA**	**RMA**	**MAS 5.0**	**MAS5.0_ _noA calls**	**PLIER**	**PLIER16**	**Plier16_ _no A calls**
**Crude MCMH**							
GO:BP							
BMD	79.34	69.48	62.67	62.67	61.69	61.69	61.69
BMDL	49.80	43.77	41.86	41.86	41.36	41.36	41.36
Reactome							
BMD	79.36	81.32	71.88	71.86	79.21	70.21	79.21
BMDL	49.80	50.42	47.15	47.15	49.59	49.59	49.59
**MCMH**							
GO:BP							
BMD	69.45	79.21	58.84	58.84	95.95	95.95	95.95
BMDL	46.21	49.59	40.76	40.76	65.58	65.58	65.58
Reactome							
BMD	223.52	284.71	174.02	174.02	271.37	264.09	264.09
BMDL	155.45	186.10	118.79	118.79	172.32	172.40	172.40
**p-toluidine**							
GO:BP							
BMD	2.88	3.00	0.33	0.33	2.82	5.29	5.29
BMDL	1.12	1.12	0.07	0.07	1.01	2.13	2.13
Reactome							
BMD	5.32	8.09	3.33	5.83	2.85	15.77	15.77
BMDL	1.73	4.89	0.66	2.52	0.98	10.99	10.99
**DMPT - liver**							
GO:BP							
BMD	1.47	1.72	1.04	1.04	1.02	1.36	1.36
BMDL	0.50	0.96	0.34	0.34	0.30	0.42	0.42
Reactome							
BMD	3.44	4.24	2.62	2.62	2.43	5.17	6.62
BMDL	1.79	2.05	1.10	1.10	0.94	1.59	3.04
**PPH-kidney**							
GO:BP							
BMD	22.65	53.35	63.40	63.40	118.53	176.02	176.02
BMDL	4.20	16.40	11.26	11.26	75.35	120.34	120.34
Reactome							
BMD	54.51	183.97	243.13	243.13	201.67	180.81	180.81
BMDL	8.58	120.87	104.12	152.92	116.43	120.34	120.34

Supplemental table 3: GO:BP ontologies and Reactome Pathways with lowest median BMD or BMDL

Accession code	Name
GO:0002933	Lipid hydroxylation
GO:0006084	Acetyl-CoA metabolic process
GO:0006090	Pyruvate metabolic process
GO:0007040	Lysosome organization
GO:0007062	Sister chromatid cohesion
GO:0009108	Coenzyme biosynthetic process
GO:0032026	Response to magnesium ion
GO:0034502	Protein localization to chromosome
GO:0035384	Thioester biosynthetic process
GO:0042130	Negative regulation of T cell proliferation
GO:0043588	Skin development
GO:0043651	Linoleic acid metabolic process
GO:0044246	Regulation of multicellular organismal metabolic process
GO:0048266	Behavioral response to pain
GO:0051445	Regulation of meiotic cell cycle
GO:0061051	Positive regulation of cell growth involved in cardiac muscle cell development
GO:0070141	Response to UV-A
GO:0070206	Protein trimerization
GO:0070207	Protein homotrimerization
GO:0070988	Demethylation
GO:0070989	Oxidative demethylation
GO:0071616	Acyl-CoA biosynthetic process
GO:0080171	Lytic vacuole organization
GO:0090207	Regulation of triglyceride metabolic process
GO:1902229	Regulation of intrinsic apoptotic signaling pathway in response to DNA damage
GO:1904705	Regulation of vascular smooth muscle cell proliferation
GO:2000727	Positive regulation of cardiac muscle cell differentiation
R-RNO-1296072	Voltage gated Potassium channels
R-RNO-141424	Amplification of signal from the kinetochores
R-RNO-141444	Amplification of signal from unattached kinetochores via a MAD2 inhibitory signal
R-RNO-1483206	Glycerophospholipid biosynthesis
R-RNO-156580	Phase II - Conjugation of compounds
R-RNO-159418	Recycling of bile acids and salts
R-RNO-163125	Post-translational modification: synthesis of GPI-anchored proteins
R-RNO-163685	Integration of energy metabolism
R-RNO-1638091	Heparan sulfate/heparin (HS-GAG) metabolism
R-RNO-174154	APC/C:Cdc20 mediated degradation of Securin
R-RNO-174154	APC/C:Cdc20 mediated degradation of Securin
R-RNO-174178	APC/C:Cdh1 mediated degradation of Cdc20 and other APC/C:Cdh1 targeted proteins in late mitosis/early G1
R-RNO-174184	Cdc20:Phospho-APC/C mediated degradation of Cyclin A
R-RNO-176417	Phosphorylation of Emi1
R-RNO-179419	APC:Cdc20 mediated degradation of cell cycle proteins prior to satisfation of the cell cycle checkpoint
R-RNO-2024096	HS-GAG degradation
R-RNO-2187338	Visual phototransduction
R-RNO-382556	ABC-family proteins mediated transport
R-RNO-5173105	O-linked glycosylation
R-RNO-5663220	RHO GTPases Activate Formins
R-RNO-69618	Mitotic Spindle Checkpoint

Accession code	Name
R-RNO-75105	Fatty acyl-CoA biosynthesis
R-RNO-75105	Fatty acyl-CoA biosynthesis
R-RNO-77289	Mitochondrial Fatty Acid Beta-Oxidation
R-RNO-8878166	Transcriptional regulation by RUNX2
R-RNO-8978868	Fatty acid metabolism

3. Supplemental figures

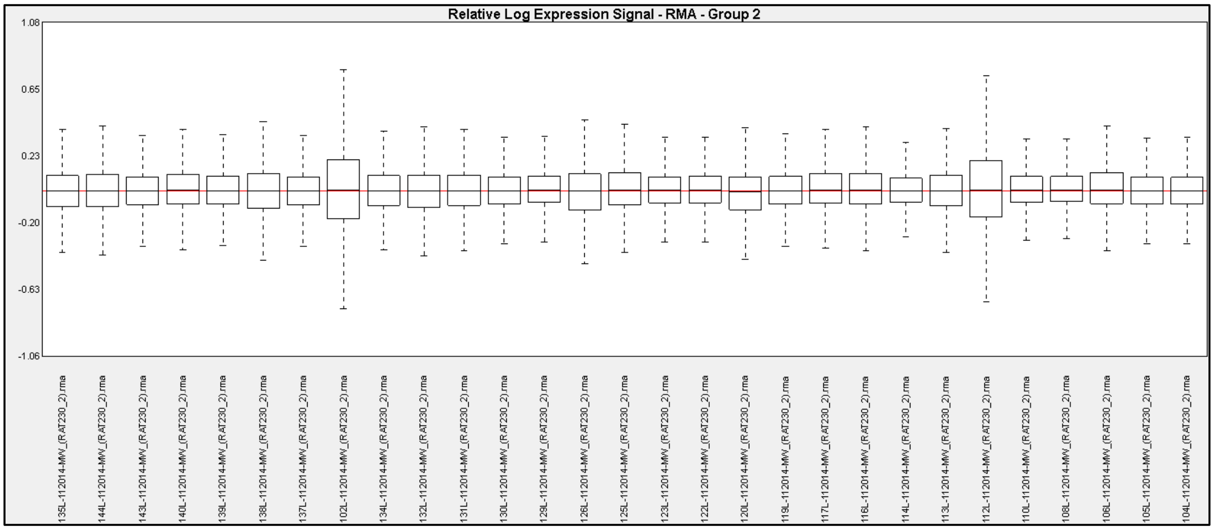

Supplemental figure 1 RLE boxplots of expression data for crude MCHM normalized by RMA before removing low-quality chips 102L and 112 L.

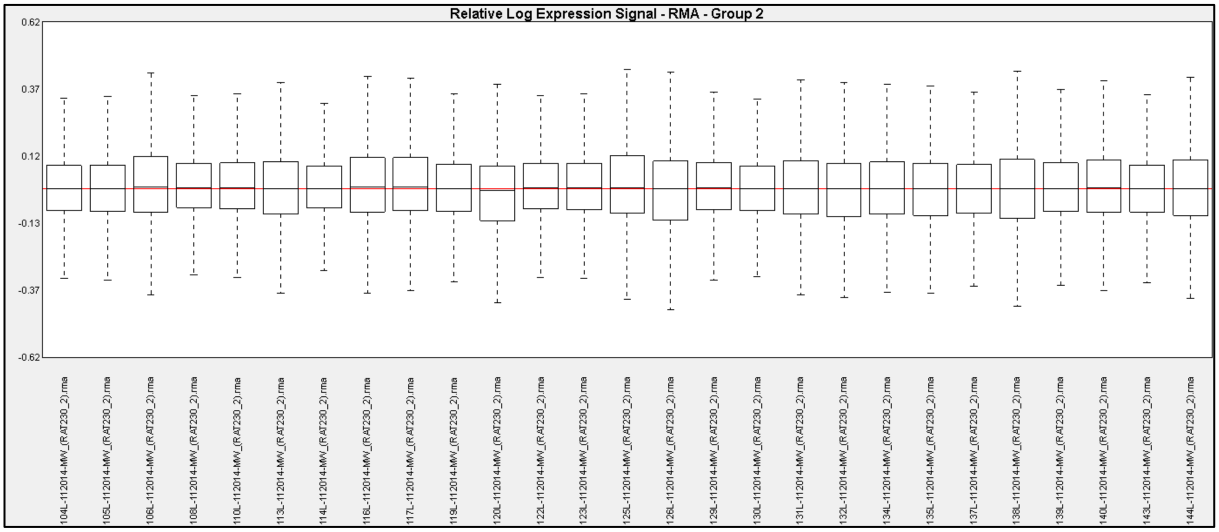

Supplemental figure 2 RLE boxplots of expression data for crude MCHM normalized by RMA after removing low-quality chips 102L and 112 L.

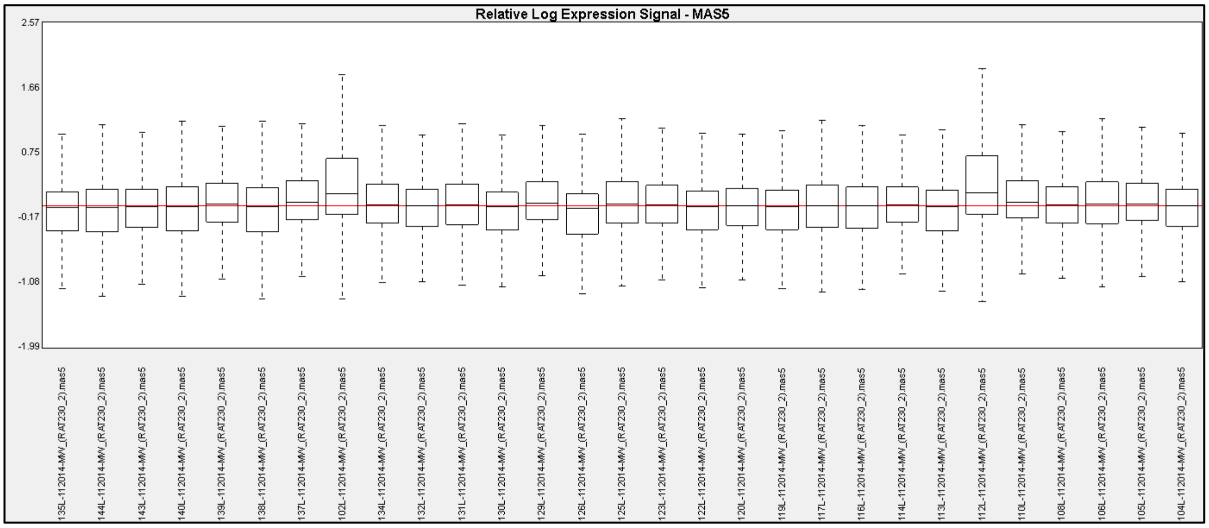

Supplemental figure 3 RLE boxplots of expression data for crude MCHM normalized by MAS 5.0 method before removing low-quality chips 102L and 112 L.

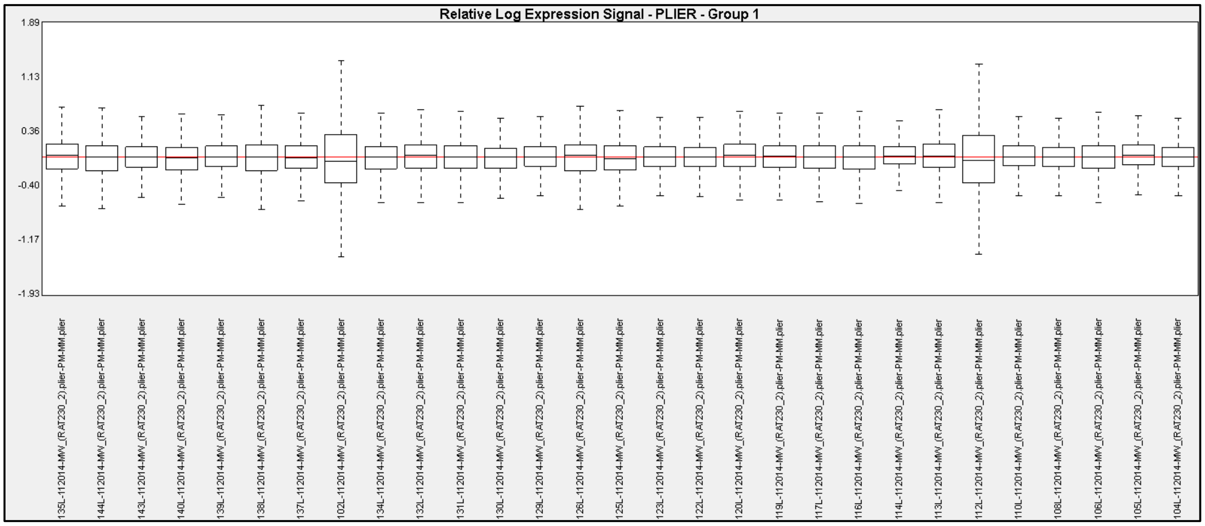

Supplemental figure 4 RLE boxplots of expression data for crude MCHM normalized by PLIER before removing low-quality chips 102L and 112 L.

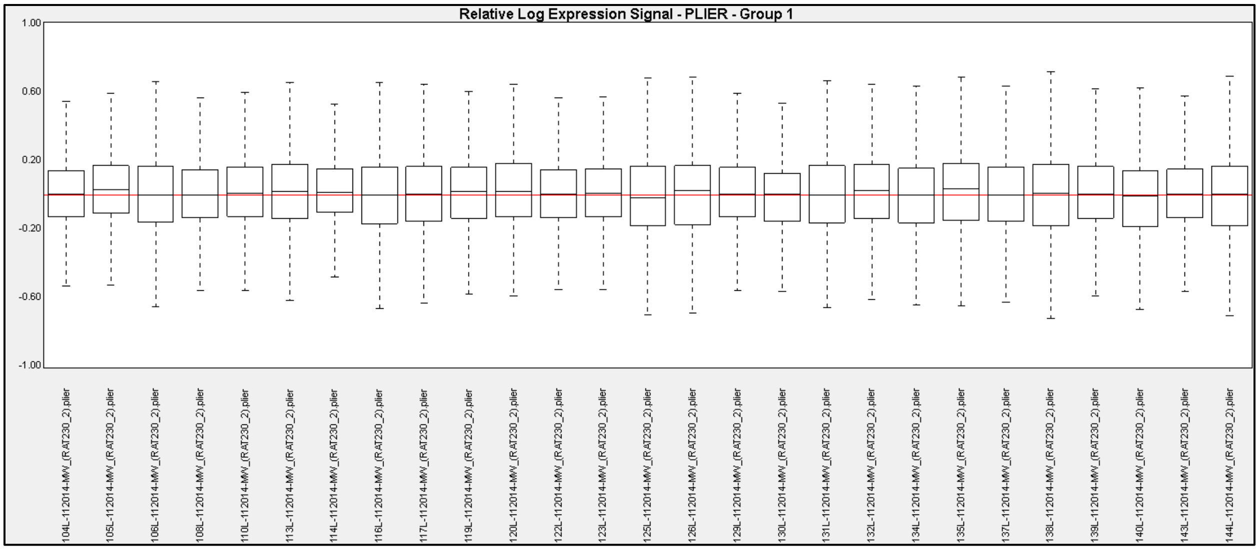

Supplemental figure 5 RLE boxplots of expression data for crude MCHM normalized by PLIER after removing low-quality chips 102L and 112 L.

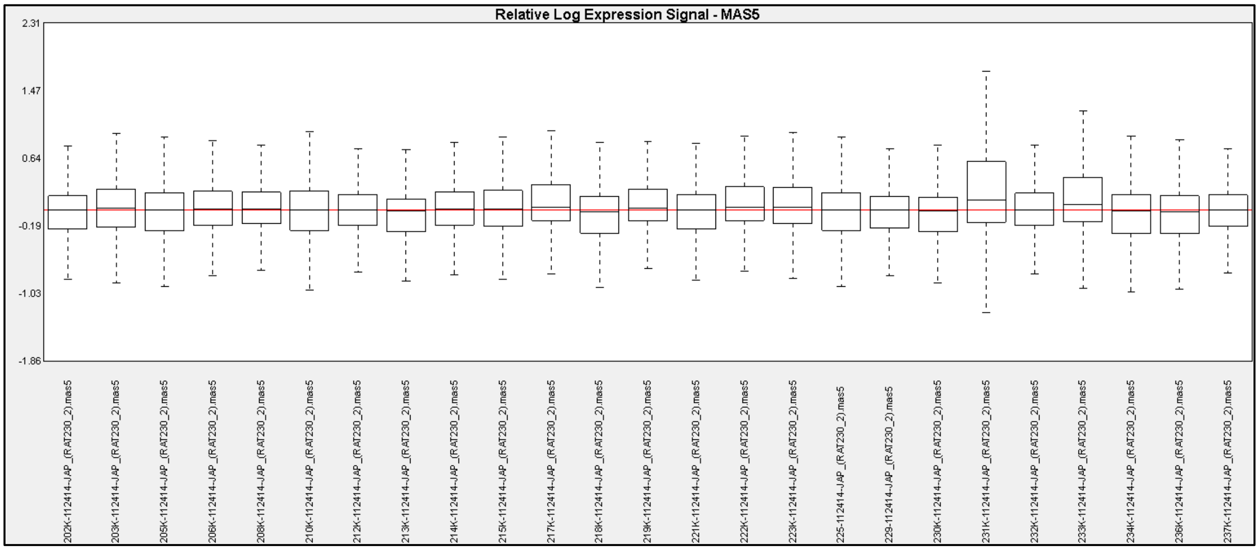

Supplemental figure 6 RLE boxplots of expression data for PPH normalized by MAS 5.0 before removing low-quality chip 231K.

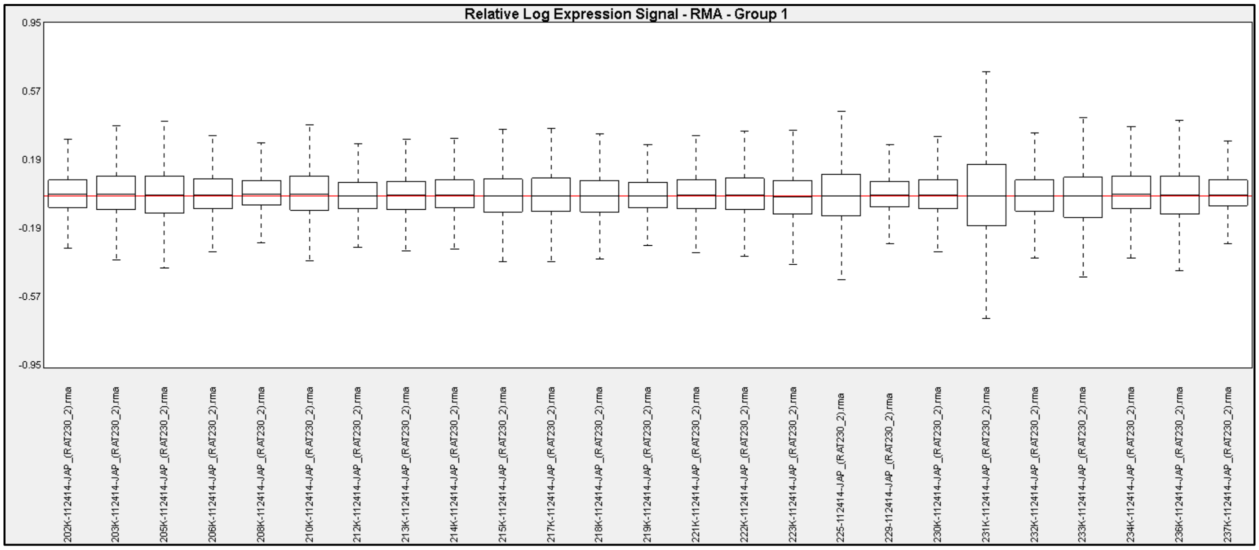

Supplemental figure 7 RLE boxplots of expression data for PPH normalized by RMA before removing low-quality chip 231K.

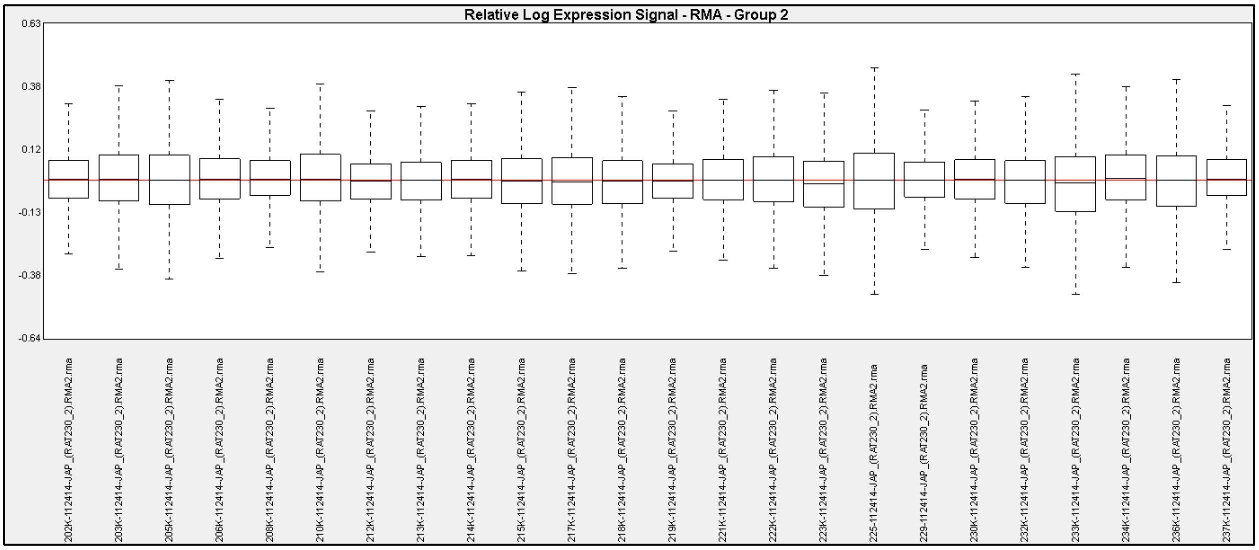

Supplemental figure 8 RLE boxplots of expression data for PPH normalized by RMA after removing low-quality chip 231K.

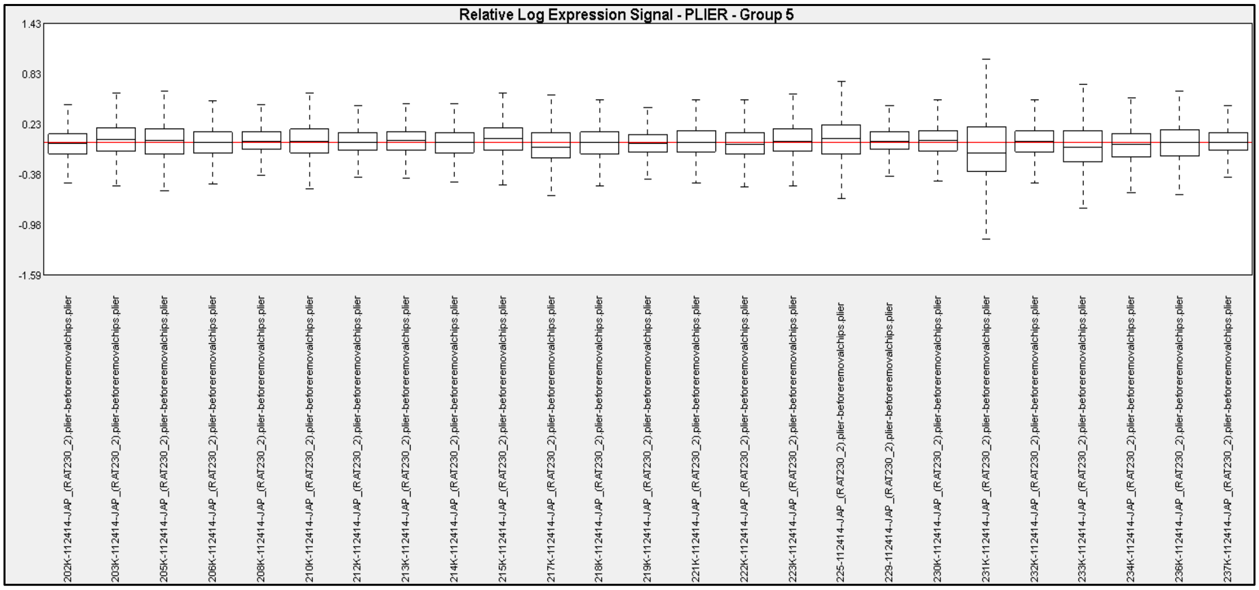

Supplemental figure 9 RLE boxplots of expression data for PPH normalized by PLIER before removing low-quality chips 217K, 225K, 231K and 233K.

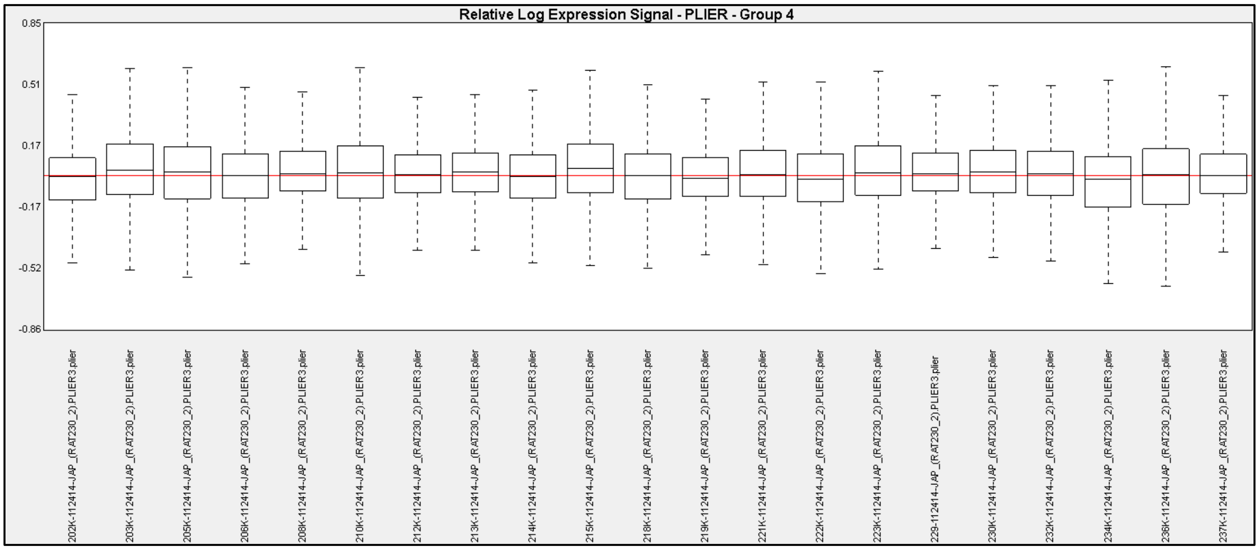

Supplemental figure 10 RLE boxplots of expression data for PPH normalized by PLIER after removing low-quality chips 217K, 225K, 231K and 233K.

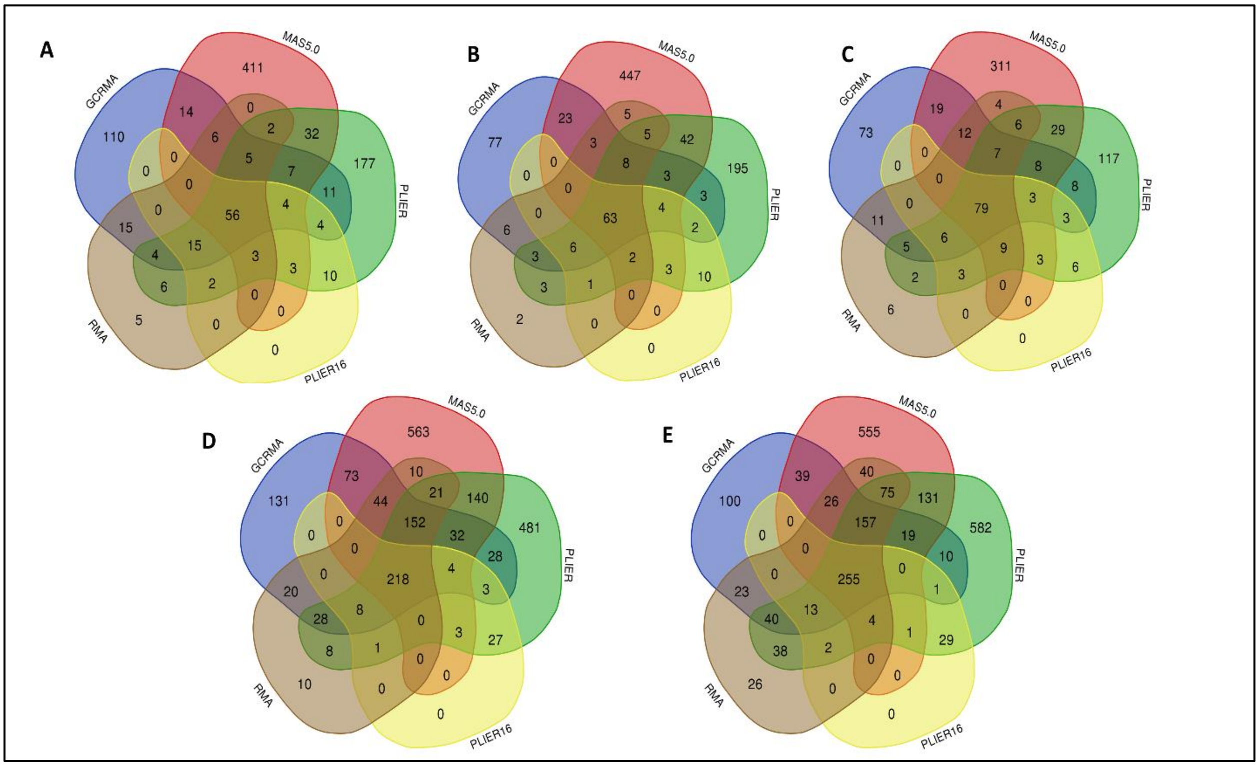

Supplemental figure 11 Numbers of differentially expressed genes identified for chemical-treatment pairs by different normalization methods depicted by Venn diagrams. A – crude MCHM/liver; B- neat MCHM/liver; C – PPH/kidney; D – DMPT/liver; E – p-toluidine/liver. Only GCRMA, RMA, MAS5.0, PLIER and PLIER16 are shown to enable visualization (normalization methods MAS5.0_noA and PLIER16_noA identify subsets of DEG found by MAS5.0 and PLIER16, respectively).

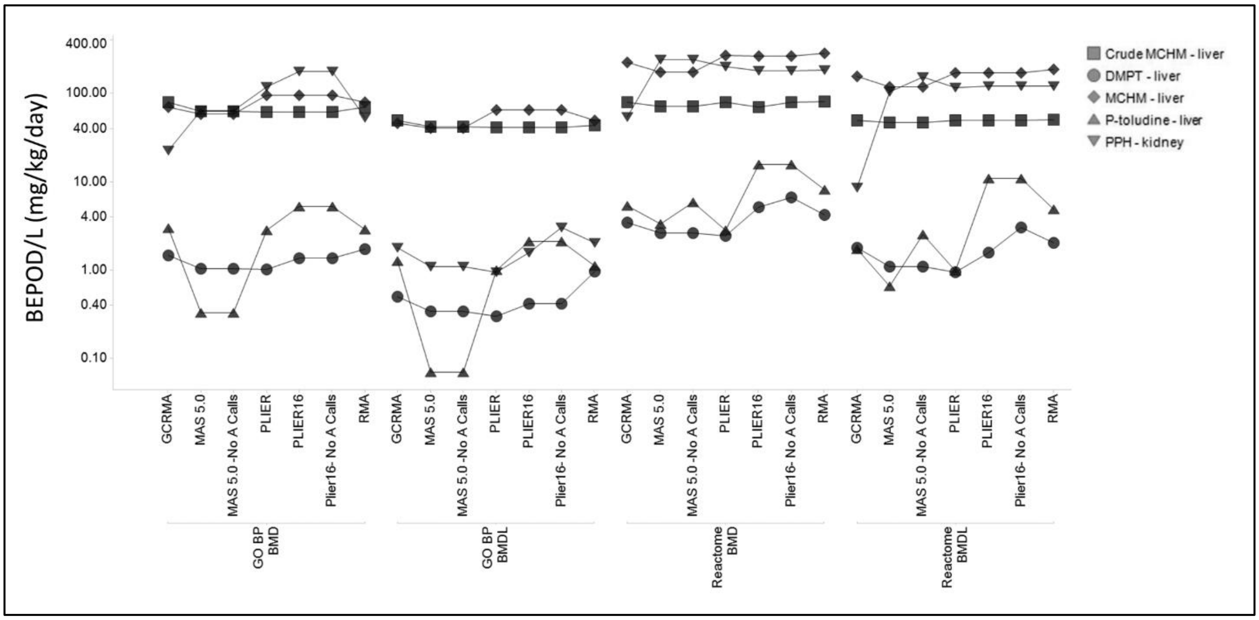

Supplemental figure 12 Lowest median BMD (BEPOD) and BMDL (BEPOD/L) values for different gene sets (GO:BP or Reactome) and normalizations (GCRMA, MAS5.0, MAS5.0_noA calls, PLIER, PLIER16, PLIER16_noA calls and RMA).
