# Supplementary figures and images for: The sensitivity of transcriptomics BMD modeling to the methods used for microarray data normalization
Source: PLoS One. 2020 May 15;15(5):e0232955. doi: 10.1371/journal.pone.0232955 (PMC7228135; doi:10.1371/journal.pone.0232955)

## Slide 1
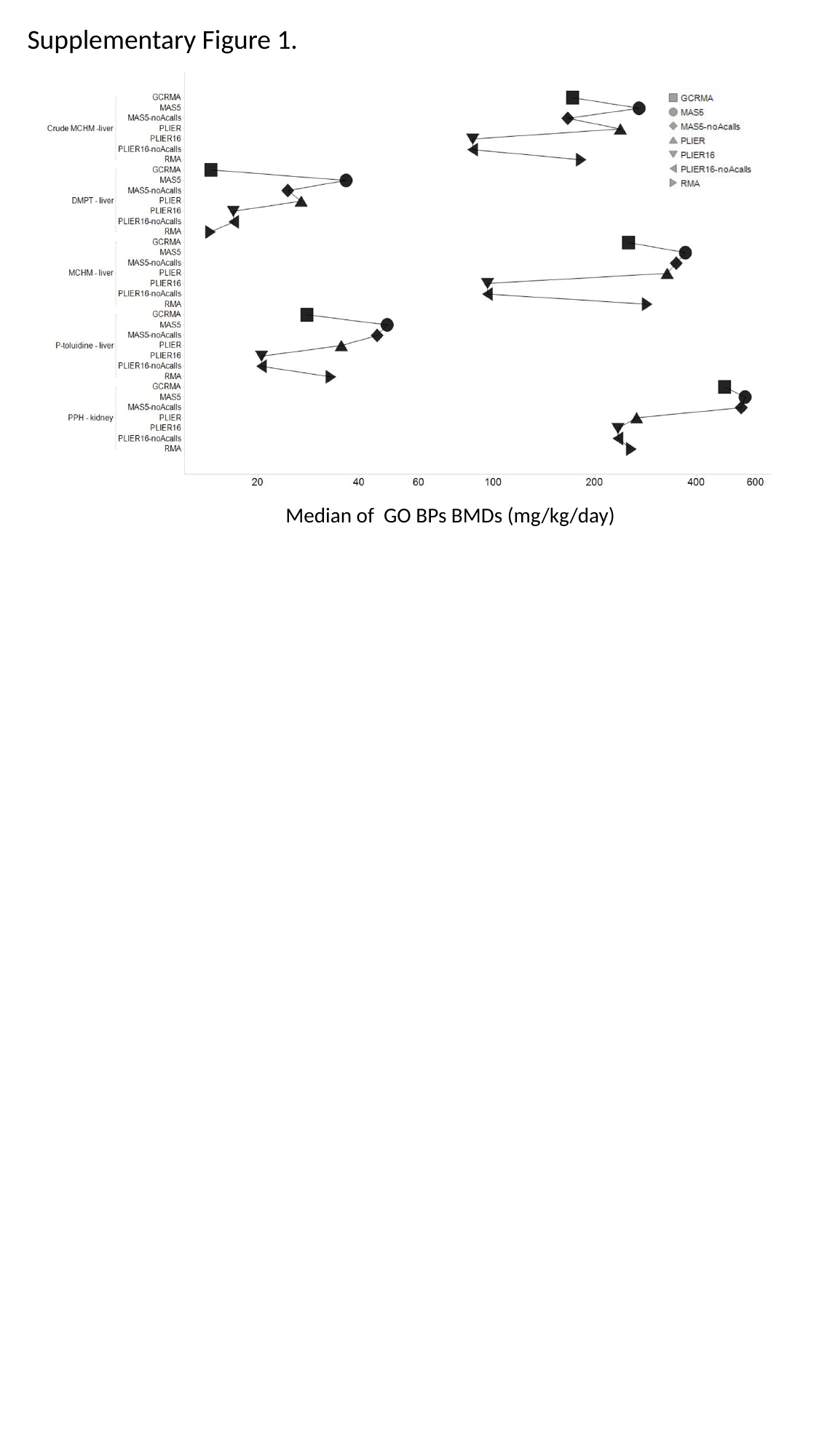

Supplementary Figure 1.
Median of GO BPs BMDs (mg/kg/day)

Supplement: S1 Fig — (PPTX) [file pone.0232955.s003.pptx]

## Slide 1
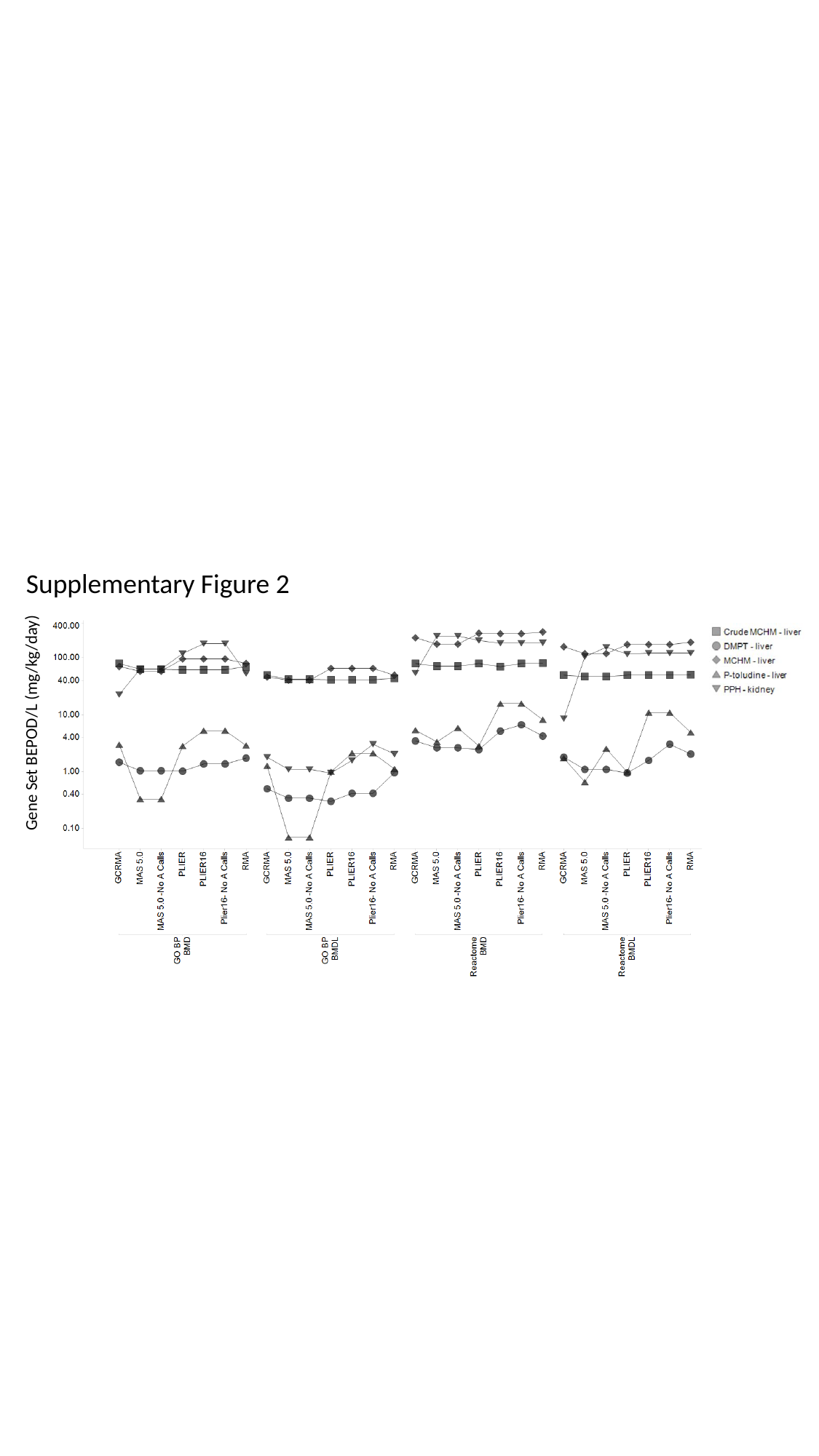

Supplementary Figure 2
Gene Set BEPOD/L (mg/kg/day)

Supplement: S2 Fig — (PPTX) [file pone.0232955.s004.pptx]
